# Reprogramming of Adult Retinal Müller Glial Cells into Human-Induced Pluripotent Stem Cells as an Efficient Source of Retinal Cells

**DOI:** 10.1155/2019/7858796

**Published:** 2019-07-15

**Authors:** Amélie Slembrouck-Brec, Amélie Rodrigues, Oriane Rabesandratana, Giuliana Gagliardi, Céline Nanteau, Stéphane Fouquet, Gilles Thuret, Sacha Reichman, Gael Orieux, Olivier Goureau

**Affiliations:** ^1^Sorbonne Université, INSERM, CNRS, Institut de la Vision, F-75012 Paris, France; ^2^Biologie, Ingénierie et Imagerie de la Greffe de Cornée, EA2521, Faculté de Médecine, Université Jean Monnet, Saint-Etienne, France

## Abstract

The reprogramming of human somatic cells to induced pluripotent stem cells (iPSCs) has broad applications in regenerative medicine. The generation of self-organized retinal structures from these iPSCs offers the opportunity to study retinal development and model-specific retinal disease with patient-specific iPSCs and provides the basis for cell replacement strategies. In this study, we demonstrated that the major type of glial cells of the human retina, Müller cells, can be reprogrammed into iPSCs that acquire classical signature of pluripotent stem cells. These Müller glial cell-derived iPSCs were able to differentiate toward retinal fate and generate concomitantly retinal pigmented epithelial cells and self-forming retinal organoid structures containing retinal progenitor cells. Retinal organoids recapitulated retinal neurogenesis with differentiation of retinal progenitor cells into all retinal cell types in a sequential overlapping order. With a modified retinal maturation protocol characterized by the presence of serum and high glucose levels, our study revealed that the retinal organoids contained pseudolaminated neural retina with important features reminiscent of mature photoreceptors, both rod and cone subtypes. This advanced maturation of photoreceptors not only supports the possibility to use 3D retinal organoids for studying photoreceptor development but also offers a novel opportunity for disease modeling, particularly for inherited retinal diseases.

## 1. Introduction

Human pluripotent stem cells (PSCs) represent a valuable tool to study human neuronal development and neurodegenerative diseases and to develop future stem cell-based therapies [1]. As concerns the retina, both human embryonic stem cells (ESCs) and induced pluripotent stem cells (iPSCs) have been shown to be able to produce retinal cells [2–7], including retinal ganglion cells, photoreceptors, and retinal pigmented epithelial (RPE) cells, corresponding to the major cell types affected in the most common retinal degenerative diseases. The differentiation of human PSCs toward the retinal lineage has evolved considerably in the last few years, and several innovative protocols allowing the self-formation of 3D retinal organoids have been reported [8–16].

Many types of somatic cells, such as skin fibroblasts, blood cells, keratinocytes, or urine-derived cells, have been successfully used for reprogramming and the production of human iPSCs [1, 17]. Due to their high availability through noninvasive and routine sampling in clinical settings, blood and urine-derived cells have been considered as a preferred source for reprogramming. Nevertheless, independently of the initial somatic identity of reprogrammed cells, human iPSCs can be guided to differentiate into retinal organoids with relatively similar efficiency using different retinal differentiation protocols [11, 18–21]. Recently, Wang et al. reprogrammed four different neuronal cell types (rod photoreceptors, cone photoreceptors, bipolar cells, and amacrine/horizontal cells) from early and late postnatal mouse retina into iPSCs and further differentiated them into retinal organoids [22]. Mouse Müller glial cells (MGCs), which originate from the same pool of retinal progenitors as retinal neurons, can also be reprogrammed to pluripotency with high efficiency and can be guided to differentiate into retinal organoids [22]. Even though no data regarding the reprogramming of human retinal cells has been reported, human glial cells such as astrocytes isolated from cerebellar tissue can be efficiently reprogrammed into iPSCs and further differentiated into different neural cell types [23, 24].

Here, we report the generation of iPSCs from human MGCs. By applying our multistep retinal differentiation protocol [19], we differentiated human MGC-derived iPSCs into pseudolaminated retinal organoids that contained all major retinal cell types.

## 2. Materials and Methods

### 2.1. Human Postmortem Tissue and Müller Glial Cell Cultures

Postmortem eye tissues were collected within 24 h after death from bodies donated to science (Laboratory of Anatomy, Faculty of Medicine of St-Etienne, France) in accordance with the French bioethics law. Handling of donor tissues adhered to the tenets of the Declaration of Helsinki of 1975 and its 1983 revision in protecting donor confidentiality.

The retina was dissected from globes by circumferential hemisection behind the ora serrata to gently remove the anterior segment including lens. The retina was carefully separated from the vitreous by transection of the papillary head and around the peripheral regions and transferred into a CO_2_-independent medium (Thermo Fisher Scientific). After removing the major blood vessels, the retina was chopped into small fragments (<2 mm^2^) and cells dissociated as previously described [25, 26] with some modifications. After 3 washes in Ringer's solution (NaCl 155 mM; KCl 5 mM; CaCl_2_ 2 mM; MgCl_2_ 1 mM; NaH_2_PO_4_ 2 mM; HEPES 10 mM; and glucose 10 mM), retinal fragments were dissociated with preactivated papain at 31.8 U/mg (Worthington) in Ringer's solution during 30 min at 37°C. Digestion was arrested by the addition of 1 ml of DMEM (Thermo Fisher Scientific) containing 10% of fetal bovine serum (FBS) (Thermo Fisher Scientific) and 25 *μ*g/ml of DNase1 (Sigma-Aldrich). The cells were homogeneously suspended with gentle up and down pipetting in prewarmed DMEM with 10% FBS and 10 *μ*g/ml gentamycin (MGC-medium) and seeded in 6 cm dishes previously coated with Poly-D-Lysin at 2 *μ*g/cm^2^ and Laminin at 1 *μ*g/cm^2^ (Sigma-Aldrich). Dissociated cells were incubated at 37°C in a standard 5% CO_2_/95% air incubator, and medium was left unchanged for 3 to 5 days and then renewed every 2 to 3 days. By the end of the first week in culture, large flattened glial cells were observed with few neuron-like cells scattered on the glial surface. Mitotic glial cells became confluent within 10 to 14 days after plating. At this time, cells were passaged by a brief incubation in TrypLE Express (Thermo Fisher Scientific) and banked (cryopreservation in FBS-10% DMSO) or seeded in 6-well plates previously coated with Poly-D-Lysin and Laminin for reprogramming.

### 2.2. Human Müller Glial Cell Reprogramming and Validation

Human MGCs at passage 1 were transduced using the CytoTune Sendai reprogramming vectors Oct4, Klf4, Sox2, and c-Myc (Thermo Fisher Scientific) and cultured for 6 days in the MGC-medium before plating on mitomycined human foreskin- (MHF-) seeded dishes. The day after, the MGC-medium was replaced with the iPS medium, corresponding to the ReproStem medium (ReproCELL, Ozyme) supplemented with 10 ng/ml of human recombinant FGF2 (PeproTech, France). The emergent human iPSC colonies were picked under a stereomicroscope according to their human ESC-like colony morphology and expanded on MHF feeder layers. After generation of a frozen stock, human iPSCs were cultured on MHF feeder layers and subsequently adapted to feeder-free conditions on truncated recombinant human vitronectin-coated dishes with Essential 8™ medium (both from Thermo Fisher Scientific) as previously described [19]. Cells were routinely cultured at 37°C in a standard 5% CO_2_/95% air incubator with a daily medium change and passaged with the enzyme-free Gentle cell dissociation reagent (Thermo Fisher Scientific) every week. The undifferentiated state of iPSC colonies was characterized by alkaline phosphatase expression as previously described [11]. Between twelve and sixteen passages, the clearance of the exogenous reprogramming factors and Sendai virus genome was confirmed by qPCR following the manufacturer's instructions (Thermo Fisher Scientific). At the same time, conventional cytogenetic analysis and *in vivo* pluripotency analysis by teratoma formation assay were performed as described previously [11]. The genomic integrity was confirmed by SNP genotyping (IntegraGen Genomics).

### 2.3. Retinal Differentiation and Human iPSC-Derived Retinal Cell Cultures

Retinal cell differentiation was based on our previously established protocol with adherent human iPSC [11, 27]. iPSCs derived from human MGCs cultured in Essential 8™ medium were switched in chemical-defined Essential 6™ medium (Thermo Fisher Scientific). After 2 days, iPSCs were cultured in the E6N2 medium composed of Essential 6™ medium, 1% N2 supplement (Thermo Fisher Scientific), 10 units/ml Penicillin, and 10 *μ*g/ml Streptomycin (Thermo Fisher Scientific). The medium was changed every 2-3 days. Around Day 28 (D28), identified self-formed retinal organoids were isolated, using a needle, with the surrounding cells and cultured as floating structures in the ProB27 medium supplemented with 10 ng/ml of recombinant human FGF2 (PeproTech), and half of the medium was changed every 2-3 days. The ProB27 medium is composed of chemical-defined DMEM:Nutrient Mixture F-12 (DMEM/F12, 1 : 1, L-glutamine), 1% MEM nonessential amino acids, 2% B27 supplement (Thermo Fisher Scientific), 10 units/ml Penicillin, and 10 *μ*g/ml Streptomycin. At D35, retinal organoids were cultured in the absence of FGF2 in the ProB27 medium with 10% FBS and 2 mM of Glutamax for the next several weeks. To evaluate maturation of photoreceptors, at D84, the retinal organoids were cultured in the ProB27 medium with 2% B27 supplement without vitamin A (Thermo Fisher Scientific) until D200.

For human iPSC-derived RPE cell cultures, identified pigmented patches were cut around D42 and transferred, noted as passage 0 (P0), onto plates coated with Geltrex (Thermo Fisher Scientific). Human iPSC-derived RPE cells were expanded in the ProN2 medium composed of DMEM/F12, 1% MEM nonessential amino acids, 1% N2 supplement, 10 units/ml Penicillin, and 10 *μ*g/ml Streptomycin and passaged or banked as previously described [19, 27].

### 2.4. Cryosection

For cryosectioning, retinal organoids were fixed for 15 min in 4% paraformaldehyde (PAF) at 4°C and washed in phosphate-buffered saline (PBS). Structures were incubated at 4°C in PBS/30% sucrose (Sigma-Aldrich) solution during at least 2 hrs and embedded in a solution of PBS, 7.5% gelatin (Sigma-Aldrich), 10% sucrose, and frozen in isopentane at -50°C. 10 *μ*m thick cryosections were collected in two perpendicular planes.

### 2.5. Immunostaining and Imaging on Retinal Sections and Dissociated Cells

Dissociated cells were fixed with 4% PAF in PBS for 10 min before immunostaining. Sections and fixed dissociated cells were washed with PBS; nonspecific binding sites were blocked for 1 hr at room temperature with a PBS solution containing 0.2% gelatin and 0.1% Triton X-100 (blocking buffer) and then overnight at 4°C with the primary antibody (Table S1) diluted in blocking buffer. Slides were washed three times in PBS with 0.1% Tween and then incubated for 1 hr at room temperature with appropriate secondary antibodies conjugated with either Cy3, Alexa Fluor 488, 594, or 647 (Interchim) diluted at 1 : 600 in blocking buffer with 4′,6-diamidino-2-phenylindole (DAPI) diluted at 1 : 1000 to counterstain nuclei. Fluorescent staining signals were captured with an Olympus FV1000 confocal microscope.

### 2.6. Retinal Organoid Immunostaining-Clearing and Imaging

Retinal organoids were fixed with 4% PAF in PBS for 30 to 60 min before immunostaining with a PBS solution containing 0.2% gelatin, 0.5% Triton X-100, and 0.01% thimerosal (Sigma-Aldrich) as previously described [11]. Samples were next submitted to the 3D imaging of solvent-cleared organ (3DISCO) clearing procedure [19]. 3D imaging was performed with an inverted confocal microscope (Olympus FV1200) with numerical objectives for high-resolution imaging. Images, 3D volume, and movies were generated with Imaris x64 software (version 7.6.1, Bitplane) using the “snapshot” and “animation” tools.

### 2.7. RNA Extraction and TaqMan Assay

Total RNAs were extracted using the NucleoSpin RNA II kit (Macherey-Nagel) and cDNA synthesized using the QuantiTect reverse transcription kit (Qiagen) following the manufacturer's recommendations. qPCR analysis was performed with custom TaqMan^®^ Array 96-Well Fast plates (Thermo Fisher Scientific) according to the manufacturer's protocol. All primers and MGB probes labeled with FAM for amplification were purchased from Thermo Fisher Scientific (Table S2). Results were normalized against 18S, and quantification of gene expression was based on the deltaCt method in minimum three independent biological experiments.

### 2.8. Phagocytosis Assay

Photoreceptor outer segments (POS) phagocytosis assay was performed as previously described [19]. Briefly, four weeks after plating confluent, human iPSC-derived RPE cells were challenged for 3 hrs with 1 × 10^6^ FITC-labeled POS before the detection of surface-bound and internalized FITC-POS particles. For exclusive detection of internalized particles, the fluorescence of surface-bound FITC-POS was selectively quenched by incubation in 0.2% trypan blue before cell fixation in ice-cold methanol. Following rehydration, cells were incubated with DAPI for nuclei counterstaining. Fluorescent signals were quantified with the Infinite M1000 Pro (Tecan). The immortalized rat RPE cell line RPE-J was used as a positive control for phagocytic activity.

## 3. Results

### 3.1. Derivation of iPSCs from Human Müller Cells

**Figure 1:**
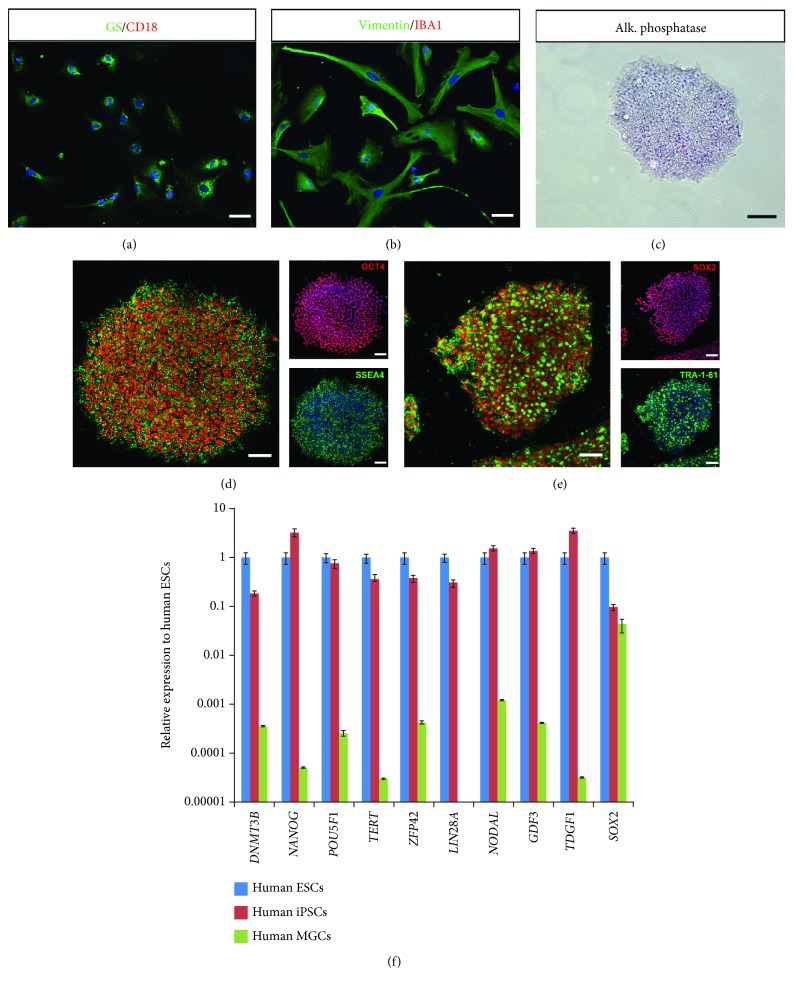
Derivation and characterization of iPSCs from human MGCs. (a-b) Characterization of human MGCs by immunostaining with glial (GS and Vimentin) and microglial (CD18 and Iba1) markers. Nuclei were counterstained with DAPI (blue). Note the absence of staining with microglial markers as expected. (c) Positive alkaline phosphatase staining of human iPSC-5f derived from human MGCs at P10. (d-e) Immunofluorescence of pluripotency markers (SSEA4, OCT4, TRA1-81, and SOX2) for iPSC-5f at P15. (f) qRT-PCR analysis of pluripotency and self-renewal markers in human ESCs, iPSC-5f, and human MGCs. Data are normalized to human ESCs (scale bars: a, b, d, e, 60 *μ*m; c, 200 *μ*m).

Retinal cells were dissociated from human postmortem retina and were seeded into tissue culture plates and left to develop as monolayer culture for one week in culture conditions previously described to favor glial cell culture while eliminating other cell types [25]. After one passage, immunostaining of the cells with anti-Vimentin and anti-Glutamine Synthase (GS) antibodies revealed homogenous labeling throughout the cytoplasm of the cells, consistent with a MGC phenotype (Figures 1(a) and 1(b)). In contrast, markers of microglia and monocytes/macrophages such as ionized calcium-binding adaptor molecule 1 (Iba1) and CD18 were absent (Figures 1(a) and 1(b)). We investigated the potential of human MGCs to be reprogrammed into PSCs using the four reprogramming factors OCT3/4, SOX2, CMYC, and KLF4 delivered using nonintegrative Sendai viruses previously used for dermal fibroblast reprogramming [28]. Emerging iPSC colonies were detectable between 10 and 15 days after transduction, and around 10 colonies were picked up and expanded for at least 5 passages before being adapted in feeder-free conditions (vitronectin coating). Expanded human iPSC colonies displayed alkaline phosphatase activity as shown in Figure 1(c) for one clone (iPSC line-5f). Immunofluorescence analysis of human iPSC line-5f revealed the coexpression of transcription factors OCT4 and SOX2, and surface markers SSEA4 and TRA1-81 (Figures 1(d) and 1(e)), characteristic of pluripotent stem cells. RT-qPCR revealed that the expression of pluripotency genes markedly increased over the respective human MGC population and was similar with that seen in human ESCs (Figure 1(f)).

Human iPSC line-5f could be differentiated *in vivo* into derivatives of all three germ layers, as shown by teratoma formation in NSG mouse (Figures S1A-S1B) and exhibited a normal karyotype after 15 passages (Figure S1C). The clearance of the vectors and the exogenous reprogramming factor genes was confirmed by qPCR after 15 passages (Figure S1D). Furthermore, genomic integrity of the iPSC line-5f was confirmed by SNP genotyping (Figure S1E).

### 3.2. Induction of Human MGC-Derived iPSCs toward Retina Cell Fates

**Figure 2:**
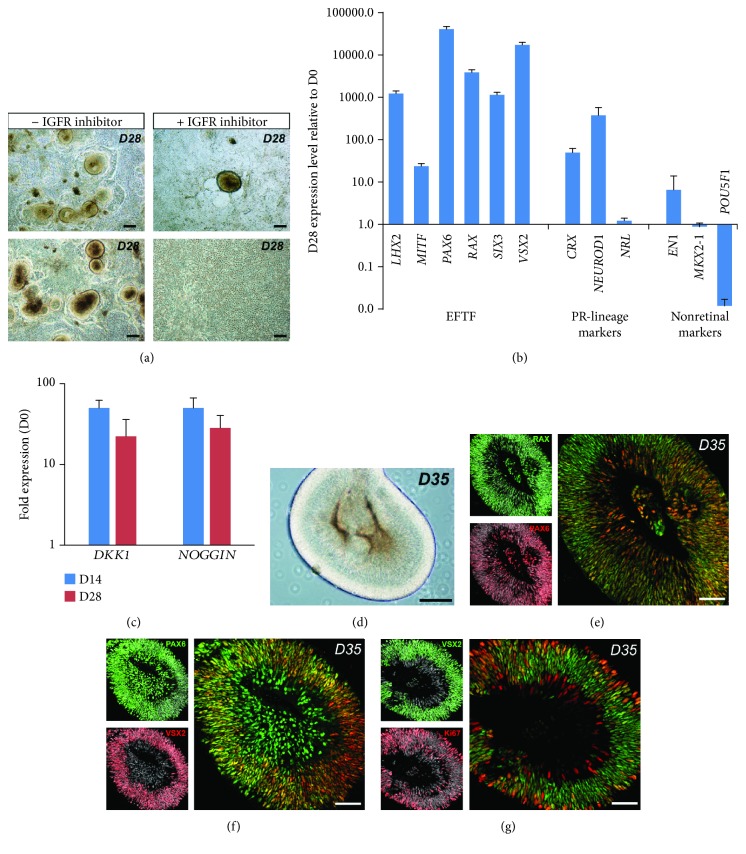
Commitment of human MGC-derived iPSCs into retinal lineage. (a) Examples of self-forming neuroepithelial-like structures derived from iPSC-5f at D28 and involvement of IGF-1/insulin signaling in neuroepithelium formation during iPSC differentiation. (b) qRT-PCR analysis of eye-field transcription factors (EFTF), photoreceptor-restricted lineage markers, and nonretinal markers in neuroepithelial-like structures at D28. Data are normalized to iPSC-5f at D0. (c) qRT-PCR analysis of NOGGIN and DKK1 in differentiating iPSC-5f at D14 and D28. Data are normalized to iPSC-5f at D0. (e-g) Immunofluorescence costaining of cryosections from neuroepithelial-like structures at D35 for RAX and PAX6 (e), PAX6 and VSX2 (f), or VSX2 and Ki67 (g). Nuclei were counterstained with DAPI (gray) (scale bars: a and d, 200 *μ*m; e, f, g, 100 *μ*m).

Based on our retinal differentiation protocol in xeno-free/feeder-free conditions [19, 27], we first evaluated the ability of overgrowing human MGC-derived iPSCs to give rise to neuroepithelial-like structures that could acquire an eye field (EF) fate. As previously reported for iPSCs derived from dermal fibroblasts, self-forming neuroepithelial-like structures can be observed about 4 weeks after the initiation of differentiation (Figure 2(a)). RT-qPCR analysis demonstrated that cells of 28-day-old (D28) structures expressed EF transcription factors, such as *LHX2*, *MITF*, *PAX6*, *RAX*, *SIX3*, and *VSX2*, while losing the expression of the pluripotency marker *POU5F1*, and showed low or no expression of forebrain and midbrain markers, such as *EN1* and *NKX2-1* (Figure 2(b)). Interestingly, the expression of transcription factors involved in the photoreceptor lineage, such as *CRX*, *NRL*, and *NEUROD1*, was also detected (Figure 2(b)). In previously published 3D induction protocols, inhibition of Wnt and BMP/TGF*β* pathways contributed to directing human PSCs to a retinal identity [7, 16]. In our protocol, RT-qPCR analysis demonstrated that differentiating human MGC-derived iPSCs expressed *DKK1* and *NOGGIN*, endogenous antagonists of Wnt and BMP, respectively (Figure 2(c)). In these conditions, inhibition of BMP/TGF and Wnt functions could occur in the absence of exogenous inhibitors, enabling the formation of neuroepithelial structures after 14 days. Activation of the insulin/insulin-like growth factor-1 signaling pathway has been also reported to contribute to directing pluripotent stem cells toward a retinal identity [13, 16]. To test the possible role of this signaling pathway, we added the pharmacological inhibitor (OSI-906) of insulin and insulin-like growth factor-1 receptor signaling to the E6N2 medium at day 2. In this condition, very rare neuroepithelial-like structures were observed after 28 days compared to the control conditions (Figure 2(a)). All these results suggested that the activation of the insulin/IGF-1 pathway and endogenous production of Wnt and BMP antagonists could explain the self-formation of these structures. After mechanical detachment at D28, structures were collected for further culture in suspension in the presence of FGF2 for one week to favor the differentiation of the neural retina (Figure 2(d)). Coexpression of RAX and PAX6 detected by immunostaining of sections from D35 organoids (after one week in floating culture) confirmed the EF identity of the organoids (Figure 2E). PAX6-positive cells located in the outer part of the developing neuroepithelium also expressed the retinal progenitor cell marker VSX2 (Figure 2(f)). At D35, many VSX2-positive cells were still mitotic as revealed by the Ki67 proliferation marker staining (Figure 2(g)), confirming the retinal progenitor cell phenotype of these cells.

### 3.3. Formation of Retinal Organoids from Human MGC-Derived iPSCs

**Figure 3:**
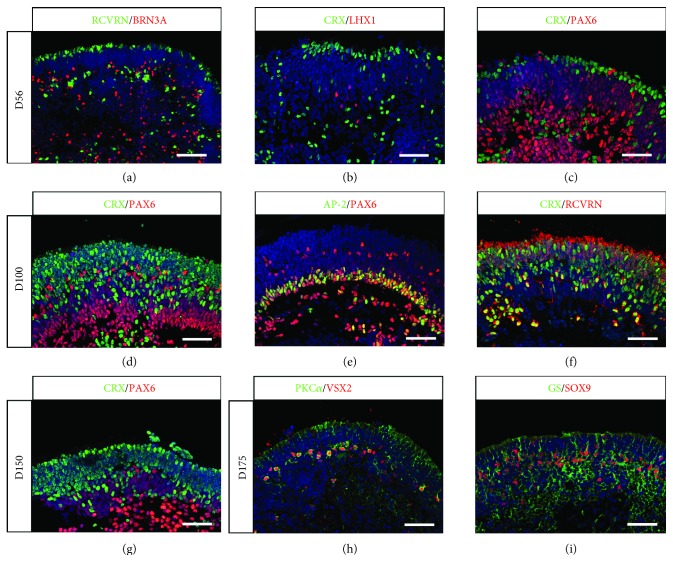
Generation of pseudolaminated retinal organoids containing all retinal cell types from human MGC-derived iPSCs. (a-f) Immunofluorescence staining of cryosections from retinal organoids at D56 (a-c) and D100 (d-f) using markers for retinal ganglion cells (BRN3A, PAX6), horizontal cells (LHX1, PAX6), amacrine cells (AP2, PAX6), and photoreceptors (CRX, RCVRN). (g-i) Immunofluorescence staining of cryosections from retinal organoids at D150 (g) and D175 (h, i) using markers for photoreceptors (CRX), bipolar cells (VSX2 and PKC*α*), and MGCs (GS and SOX9). Nuclei were counterstained with DAPI (blue) (scale bars: a, 100 *μ*m; b-i, 50 *μ*m).

During retinal development, multipotent retinal progenitors can give rise to all retinal cell types in a defined overlapping chronological order with early-born cell types like retinal ganglion cells (RGCs) and horizontal/amacrine cells and late-born cell types including bipolar cells and MGCs. Regarding photoreceptors, cones are mostly produced during early stages of retinal development, whereas rods are generated later [29, 30]. To assess the identity of differentiating retinal cells within organoids, immunohistochemistry was performed on sections after different periods in floating culture conditions, based on our previous retinal differentiation protocol (see Section 2.3 for details). At D56, BRN3A-positive RGCs were scattered throughout the organoid with a higher concentration at the innermost part of the forming neural retina (Figure 3(a)), while cells committed toward photoreceptor lineage expressing CRX and Recoverin (RCVRN) were found on the opposite side of the structure, in the external part of the retinal organoids (Figures 3(a)-3(c)). Sparse horizontal cells expressing transcription factor LHX1 can also be detected at the same time (Figure 3(b)). Immunostaining of D56 retinal organoid sections with the exclusive markers CRX for photoreceptors and PAX6 for early-born nonphotoreceptor cells (RGCs, amacrine/horizontal cells) showed that PAX6-positive neurons and CRX-positive photoreceptors started to form two distinct layers (Figure 3(c)). Interestingly, cells displaying the highest level of PAX6 were found in the most central part of the structure, while in the external part of the retinal organoid, Pax6-positive cells displayed a low level of expression of this transcription factor. As time progressed, these two layers can be more distinctly visualized, as shown by the exclusive location of CRX-expressing photoreceptors and PAX6-positive cells at D100 (Figure 3(d)) and D150 (Figure 3(g)). In addition to displaying a pseudolamination with both presumptive outer nuclear layer (ONL) and inner nuclear layer (INL), retinal organoids also showed organized PAX6-positive neurons presented toward the basal surface, where RGCs are located, and also localized in the presumptive INL (Figures 3(d) and 3(e)). Amacrine cells identified by PAX6 and AP-2 costaining formed a clear nuclear layer at D100, while sparsely distributed PAX6-positive/AP-2-negative cells in the apical part of the presumptive INL could correspond to the horizontal cells (Figure 3(e)). The population of CRX/Recoverin-positive photoreceptors congregated at the outer part of the retinal organoids (Figure 3(f)), corresponding to the presumptive ONL. Consistent with *in vivo* retinogenesis, late-born bipolar cells can be identified by costaining with PKC*α* and VSX2 antibodies (Figure 3(h)), demonstrating that our culture conditions allowed the generation of all five types of retinal neurons in organoids. Furthermore, RPCs were also able to differentiate in MGCs, as shown by the presence of cells coexpressing Glutamine Synthase (GS) and the transcription factor SOX9 in D175 retinal organoids (Figure 3(i)).

**Figure 4:**
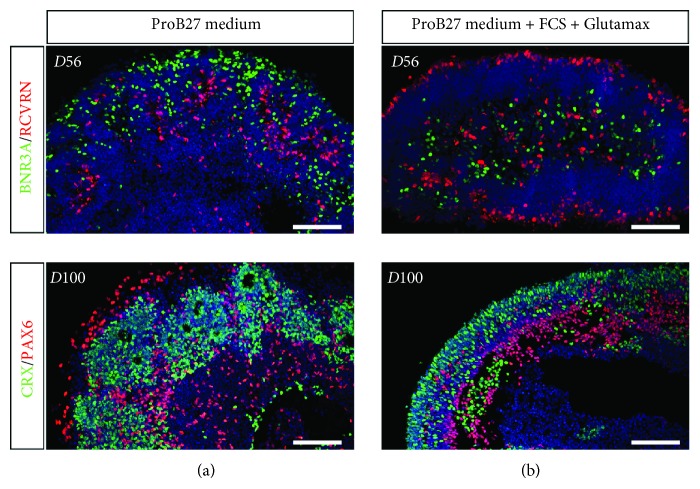
Improvement of retinal organoid lamination in the presence of FCS and Glutamax. Immunofluorescence staining of cryosectioned retinal organoids from human MGC-derived iPSCs using markers for retinal ganglion cells (BRN3A, PAX6), amacrine/horizontal cells (PAX6), and photoreceptors (CRX) after 56 and 100 days in floating cultures in the absence (a) or in the presence (b) of 10%FCS and 2 mM Glutamax in the ProB27 medium. Nuclei were counterstained with DAPI (blue) (scale bars: 100 *μ*m).

When retinal organoids from human MGC-derived iPSCs were subjected to differentiation in the ProB27 medium in the absence of FBS and Glutamax, efficient retinal differentiation was still observed with the presence of RGCs expressing BRN3A in D56 organoids and photoreceptors identified by Recoverin and CRX immunostaining in D56 and D100 organoids (Figure 4). However, organoids failed to display a continuous laminar organization with the neuroepithelial layer mostly developing into small rosettes, where the innermost cells expressed photoreceptor markers, surrounded by PAX6-positive nonphotoreceptor cells (Figure 4). This phenotype was similar to previously characterized iPSC line (hiPSC-2 clone) derived from human fibroblasts [19]. Interestingly, pseudolaminated retinal organoids can also be obtained from this hiPSC-2 line in the presence of 10%FBS and Glutamax, with similar formation of presumptive ONL, bearing CRX and Recoverin-positive photoreceptors, and presumptive INL containing PAX6-positive cells, including amacrine cells expressing AP-2 (Figure S2). Cone arrestin (CAR) immunostaining was also restricted to the presumptive ONL (Figure S2), confirming that the generation of pseudolaminated organoids was independent on the iPSC line used as a source for differentiation and was rather due to the refinement of our original retinal differentiation protocol [19, 27].

### 3.4. Maturation of Rods and Cones in Retinal Organoids

**Figure 5:**
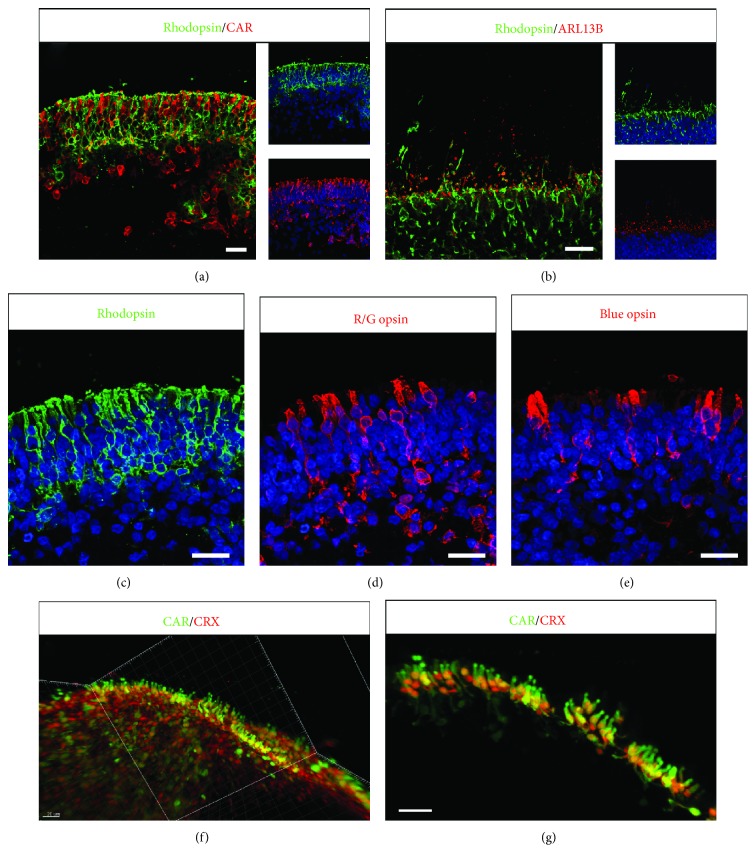
Photoreceptor development in retinal organoids from human MGC-derived iPSCs. (a-e) Immunofluorescence staining of cryosections from retinal organoids at D175 using specific photoreceptor markers to identify both rods (Rhodopsin), cones (CAR, R/G Opsin, blue Opsin), and cilia marker (ARL13B). Nuclei were counterstained with DAPI (blue). (f, g) 3D views of solvent-cleared D175 retinal organoids immunostained for Cone arrestin (CAR) and CRX. (g) High-magnification image of isolated cones from (f) (white frame) with Imaris software (scale bars: 20 *μ*m).

Next, we sought to study the maturation of photoreceptor precursors, previously identified with CRX staining (Figure 3), in the presence of FBS and Glutamax for long-term culture. Constant application of retinoic acid (RA) has been reported to inhibit cone maturation in zebrafish [31] and recently in mouse PSC-derived retinal organoids [32]. Exogenous RA was never present in our culture conditions. Retinal organoids first cultured with normal B27 supplement containing vitamin A were switched at D84 in the ProB27 medium with B27 supplement without vitamin A to prevent the formation of endogenous RA, one active metabolite of vitamin A. Rods and cones can be clearly identified either by Rhodopsin or by Cone arrestin immunostaining restricted to the apical presumptive ONL in D175 retinal organoids (Figure 5). Rhodopsin staining was distributed throughout the soma of rods and intensively stained the peripheral region of the presumptive ONL, reflecting the formation of outer segment- (OS-) like structures (Figures 5(b) and 5(c)). The ciliary protein ARL13B presented an expected punctuated labeling pattern closed to the presumptive ONL, indicating the formation of correctly positioned IS and OS-like structures (Figure 5(b)). Both short wavelength Opsin (blue Opsin) and long/medium wavelength Opsin (R/G Opsin) cones were observed with the presence of specific cone pedicle-like structures, apical to the presumptive ONL (Figures 5(d) and 5(e)). To visualize photoreceptors in 3D, we performed high-resolution confocal imaging of immunolabelled D175 whole organoids subjected to 3DISCO clearing procedure [19]. Spatial arrangement of cones characterized by the expression of CRX and cone arrestin was observed on 3D-reconstructed images with unambiguous morphological stubby bud-like cone features (Figures 5(f) and 5(g); Video S1).

### 3.5. Generation of RPE Cells from Human MGC-Derived iPSCs

**Figure 6:**
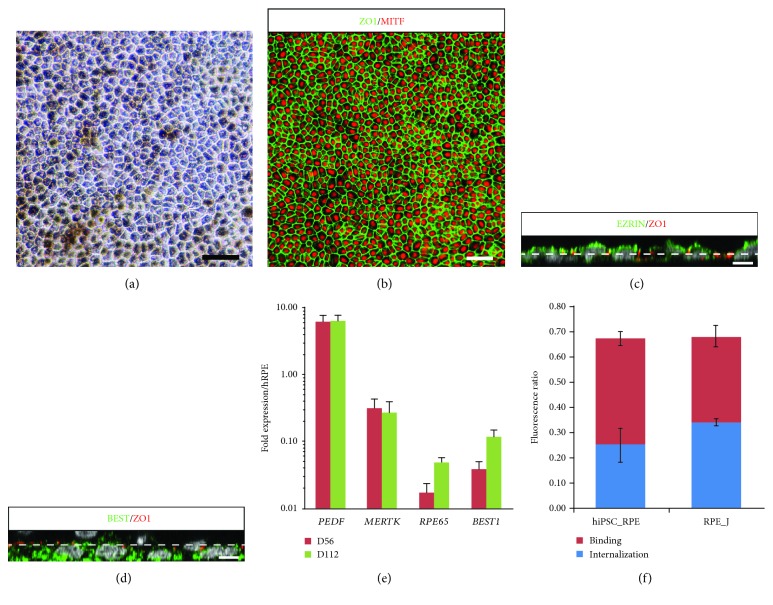
Generation of RPE cells from human MGC-derived iPSCs. (a) Phase-contrast images of RPE cells derived from iPSC-5f at passage 1 (P1), four weeks after picking. (b) ZO1 and MITF immunostaining of hiPSC-derived RPE cell monolayer four weeks after picking. (c, d) XZ views after orthogonal reconstruction of confocal stacks showing typical polarized expression of BEST1 (basal) and Ezrin (apical), four weeks after picking. Dash line mark out the apical and basolateral compartments according to ZO1 labeling. (e) qRT-PCR analysis of mature RPE markers in human iPSC-derived RPE cells at P1 and P2. Data are normalized to control RNA isolated from human adult RPE cells. (f) Evaluation of ratio of FITC/DAPI fluorescence in human iPSC-derived RPE cells at P1 and in control RPE-J cell line after 3 h incubation with FITC-labeled POS to determine RPE cell phagocytic activity; binding and uptake of POS were assayed as described Materials and Methods (scale bars: a, b, 50 *μ*m; c, d, 5 *μ*m).

After removal of retinal organoids and further differentiation of iPSC cultures in proneural medium, pigmented patches of cells appeared around D42 and could be mechanically isolated and spread onto new plates for expansion and amplification. After 4 weeks, pigmented cells formed a confluent monolayer with a cobblestone-shaped morphology (Figure 6(a)) and expressed the RPE-specific transcription factor MITF and the tight junction marker ZO-1 (Figure 6(b)). Z-stack analysis of confocal images showed the expression of Ezrin at the apical side of the cells and basolateral localization of bestrophin (Figures 6(c) and 6(d)), which is consistent with a mature polarized RPE. RT-qPCR studies demonstrated that iPSC-derived RPE cells expressed classic markers of RPE such as *PEDF*, *MERTK*, *RPE65*, and *BEST1* after long-term cultures (Figure 6(e)). We also evaluated the functionality of the iPSC-derived RPE cells by measuring the phagocytosis of fluorescent-labeled photoreceptor outer segments (POS). As shown in Figure 5(f), iPSC-derived RPE cells after one passage were able to phagocyte with an average of 37.3 ± 0.07% (mean ± SEM; *n* = 3) internalized POS within 3 hours, similar to the control rat RPE-J cell line (49.6 ± 0.02; mean ± SEM; *n* = 3).

## 4. Discussion

In this study, we generated iPSCs from a differentiated cell type isolated from normal human retina and showed that these human iPSCs derived from MGCs can be efficiently differentiated toward retinal lineage with simultaneous formation of RPE cells and retinal organoids. Specification and maturation of retinal cells generated from MGC-derived iPSCs follow the same spatial-temporal pattern observed with retinal cells derived from other human somatic-derived iPSCs and consistent with *in vivo* development.

Since all body cells seem to have the potential to become iPSCs, though at different yields, it is not surprising that glial cells from the retina, such as MGCs, can be reprogrammed into iPSCs. Furthermore, MGCs represent the most plastic cell type found in the retina. In cold-blood vertebrate, MGC population constitutes an adult retinal stem cell niche able to dedifferentiate, proliferate, and generate new retinal cells, mainly after activation of the Ascl1/Lin28 pathway following injury [33, 34]. This physiologic response is absent in mammals but *in vivo* ectopic expression of a specific combination of factors targeting mouse MGCs enabled MGCs to generate functional retinal neurons in different conditions [35, 36], confirming the latent stem cell potential of MGCs even in mammals.

Detailed examination of a variety of iPSCs has shown that these cells can retain some epigenetic memory of the cell of origin that bias their differentiation tendency toward the original cell type [37, 38]. While this phenomenon was obvious in early-passage iPSCs, the differences in epigenetics and differentiation capacities tended to be attenuated after prolonged passages [37, 38]. Other studies have shown that the cell type of origin contributed minimally to iPSC variability and that the variations in differentiation were largely attributable to donor differences rather than to the original cell type [39–41]. Even though no epigenetic characterizations have been done on the iPSCs used in this study, either derived from human MGCs or derived from dermal fibroblasts [11, 19], no major difference in the characteristics of retinal organoids has been observed. In agreement with our observation, Capowski et al. recently reported the ability of human iPSCs derived from fibroblasts or blood cells to generate retinal organoids, demonstrating the reproducibility of retinal organoid generation independently of the starting cell type [21]. A different observation has been made using mouse iPSCs derived from different retinal cell types [42]. These authors showed that rod-derived iPSCs were more efficient to differentiate into retinal structures containing all retinal cell types compared to structures obtained from iPSCs derived from fibroblasts. The authors hypothesized that epigenetic memory and especially the methylation status of the *Lhx9* gene could explain these differences. Epigenetic differences could be lower between human fibroblasts and human MGCs than between mouse fibroblasts and mouse photoreceptors. However, retinal organoids generated from mouse ESCs or iPSCs by other groups with relatively similar protocols of differentiation never reported a significant impact of the source of cells on the formation of retinal organoids [43–46].

The number of methods for generating 3D retinal cultures from human PSCs has greatly increased in the past five years [11–16, 18–21], all seeking at reproducing, despites few differences, the key steps of retinal development that are highly conserved throughout the vertebrate evolution. Morphological, cellular, and molecular analyses allow to determine the identity and the distribution of retinal cell types at different stages of organoid formation. As in many 3D protocols of differentiation, we can distinguish three successive stages in cultures of differentiating organoids. Early organoids around D35 correspond essentially to expansion of RPCs within the neuroepithelial cell layer. At intermediate stages (between D50 and D100), a clear separation between presumptive ONL and INL—corresponding to differentiation of photoreceptors and neuron populations of inner retina—could be observed in the majority of organoids. Finally, in late retinal organoids (D150-D175), an advanced stage of photoreceptor development can be obtained, revealed by the formation of IS and OS, within an expected time range regarding human development during which OS formed and elongated between fetal weeks 23 and 30 [47, 48]. However, a progressive disorganization of the inner region of the structures was observed concurrently with the RGC loss that is expected in this type of cultures in the absence of central projection targets. The requirement of RGCs for proper retinal lamination has been demonstrated in mouse and zebrafish retina, where the developmental ablation of a RGC subpopulation or interfering with the RGC migration and positioning led to a lamination defect [49, 50]. Neuronal migration and lamination play a central role in the building of the retinal laminar architecture [51]. It is likely that human iPSC-derived retinal organoids do not entirely recapitulate this highly controlled process of neuronal layering that takes place during retinogenesis. It is also important to consider that *in vivo* the inner retina is vascularized in contrast to the outer retina; thus, the absence of vasculature in retinal organoids could limit the metabolic support of the inner retina structures and explain the disorganization for long-term culture. The absence of RPE could also contribute to impaired organoid lamination. Even though our protocol allowed the generation of RPE cells from iPSCs, this monolayer of epithelial cells facing the photoreceptors *in vivo* is not present in retinal organoids as a monolayer. As reported in mice, ablation of RPE during early retinal development is accompanied by disruption of retinal lamination with formation of rosette [52].

In this study, we used FBS and Glutamax for long-term maintenance of cell survival [18] without addition of RA to the media throughout the whole differentiation process in order to try optimizing cone differentiation. Indeed, the RA signaling pathway plays an important role in photoreceptor development by promoting rod differentiation but inhibiting cone maturation in chicken and zebrafish [31, 53–55]. Recently, different groups reported that continued supplementation of RA hindered or prevented the expression of cone markers in retinal organoids derived from mouse or human iPSCs [18, 32] and that exogenous RA addition was not required to generate retinal organoids with both mature rod and cone photoreceptors [20]. Most of the recent protocols aiming at generating retinal organoids applied a relatively high concentration of RA in the culture medium in a specific time window, generally before photoreceptor maturation (around day 85-120 depending on the protocol used, yielding rod-enriched organoids) [14, 15, 21]. In our culture conditions, the precursor of RA, vitamin A, is present in B27 supplement only at early time of organoid floating cultures but not for long-term cultures (after 12 weeks), in an attempt to control the endogenous RA production during the culture. It would be interesting to investigate the expression of RA-synthesizing enzymes (Aldh1a1 and Aldh1a3) and catabolizing enzymes (Cyp26a1 and Cyp26a3) in human retinal organoids at different stages of maturation, since the transient and highly localized expression pattern of these enzymes in human fetal retina could explain the foveal patterning [55]. Recent data in mouse PSC-derived retinal organoids reported a drastic downregulation of RA metabolism enzymes (Raldh1, Raldh3, and Cyp26a1) at late differentiation stages, when cone precursors are present [32]. As reported by other groups [18, 20, 21, 56], we found that human organoids have similar distribution of cone subtypes, with less blue cones than red/green cones, as to the *in vivo* situation of human retina. The control of cone subtype specification by thyroid hormone signaling, largely described in mice [29, 30], was recently confirmed in human PSC-derived retinal organoids by elegant CRISPR/Cas9-mediated gene editing [56]. In our study, the presence of triiodothyronine (T3), the most active form of thyroid hormone in the B27 supplement, makes it possible to control the cone subtype specification in retinal organoids via the thyroid hormone receptor beta.

In summary, stem cell-derived retinal organoid technology represents a useful tool for studying the development of human retina and for modeling human retinal diseases, particularly those targeting the outer retina. With the generation of relevant cells, this innovative technology is expected to facilitate the development of cell therapies for retinal dystrophies.

## Data Availability

The data used to support the findings of this study are available from the corresponding author upon request.

## Supplementary Materials

Supplementary Materials Figure S1: additional characterization of human MGC-derived iPSC lines Figure S2: reproducibility of retinal organoid lamination and photoreceptor differentiation with a human iPSC line derived from fibroblasts. Table S1: list of antibodies used for immunohistochemistry analysis. Table S2: list of TaqMan^®^ Gene Expression ID Assays used for RT-qPCR. Movie S1: movie of a D175 retinal organoid labelled with anti-CRX (white) and Cone arrestin (red). Scale bar = 300 *μ*m.
