# Supplementary material for: Reprogramming of Adult Retinal Müller Glial Cells into Human-Induced Pluripotent Stem Cells as an Efficient Source of Retinal Cells
Source: Stem Cells Int. 2019 Jul 15;2019:7858796. doi: 10.1155/2019/7858796 (PMC6664555; doi:10.1155/2019/7858796)
Supplement: Supplementary Materials — Figure S1: additional characterization of human MGC-derived iPSC lines Figure S2: reproducibility of retinal organoid lamination and photoreceptor differentiation with a human iPSC line derived from fibroblasts. Table S1: list of antibodies used for immunohistochemistry analysis. Table S2: list of TaqMan® Gene Expression ID Assays used for RT-qPCR. Movie S1: movie of a D175 retinal organoid labelled with anti-CRX (white) and Cone arrestin (red). Scale bar = 300 μm. [file 7858796.f1.zip › Supplementary Materials.pdf]

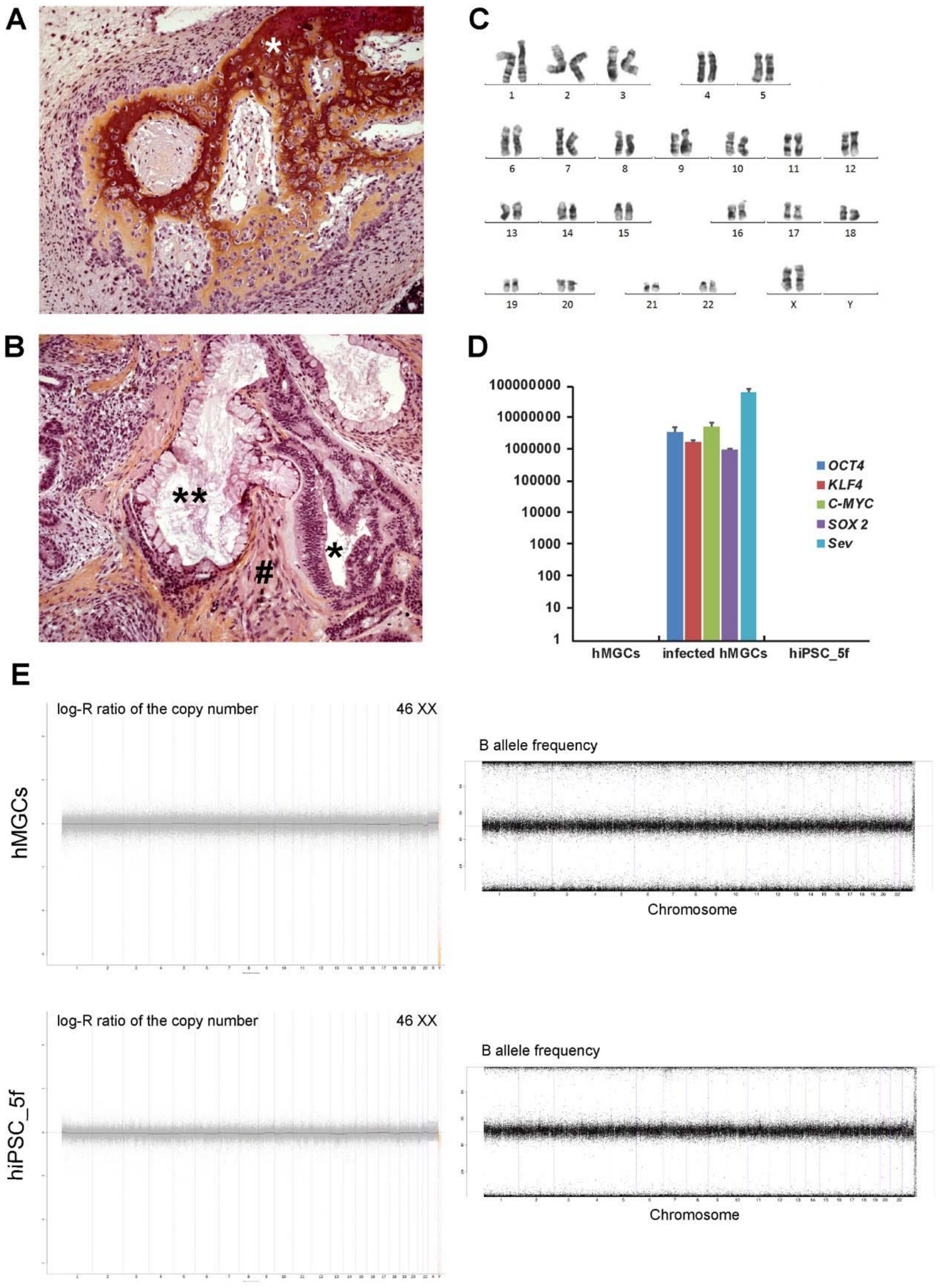

Figure S1: Additional characterization of human MGC-derived iPSC lines

(**A-B**) Histological analysis of teratomas observed in NSG mouse after subcutaneous injection of iPSC-5f; (*) bone; (*) neural tube, (**) intestinal epithelium, (#) muscular

fiber. (**C**) Karyotype analysis of human iPSC-5f. (**D**) Absence of Sendai viral ARN expression in human iPSC-5f cell line. One-week infected-MGCs were used as a positive control and data are normalized to uninfected MGCs. (**E**) SNP array analysis of human MGCs (hMGCs) and human iPSC lines (hiPSC_5f). Data are presented as the weighted log2 ratio of the copy number (Y-axis on left panels) or B allele frequency (Y-axis on right panels) and the chromosome number (X-axis).

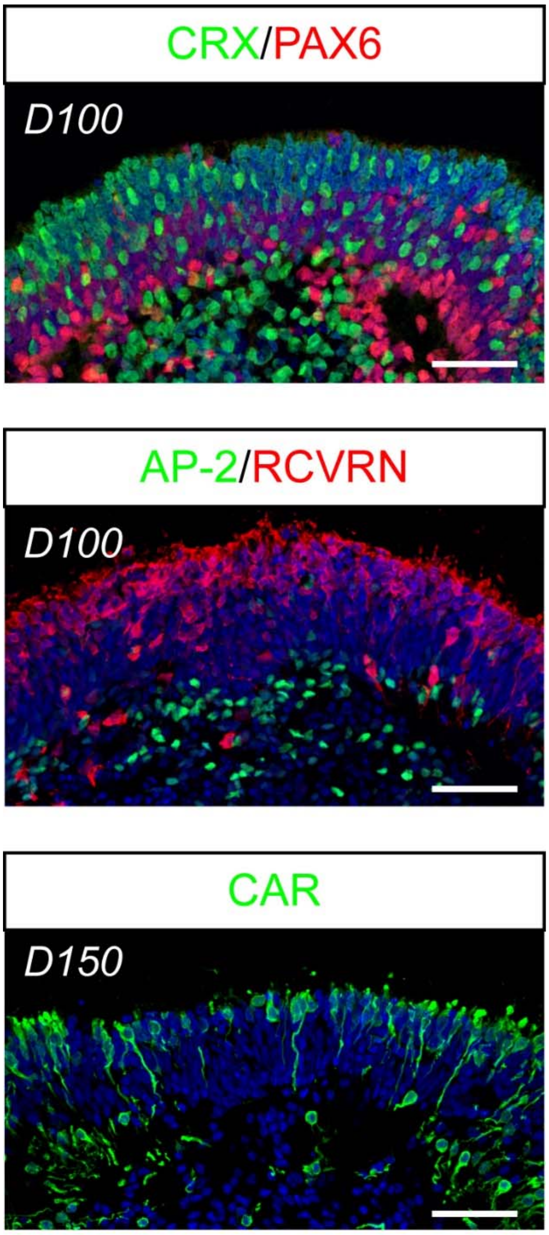

Figure S2: Reproducibility of retinal organoid lamination and photoreceptor differentiation with a human iPSC line derived from fibroblasts. Immunofluorescence staining with PAX6 (ganglion cells, amacrine/horizontal cells), AP-2 (amacrine cells), CRX and RCVRN (photoreceptors) and CAR (cones) of cryosections from D56 and D100 retinal organoids differentiated from human fibroblast-derived iPSCs (hiPSC-2 clone) in ProB27 medium supplemented with 10%FCS and 2mM Glutamax. Nuclei were counterstained with DAPI (blue). (Scale bars: 50µm).

Movie S1: Movie of a D175 retinal organoid labelled with anti-CRX (white) and Cone arrestin (red). Scale bar = 300µm. (related to Figure 5).

Table S1: List of antibodies used for immunohistochemistry analysis

Antigen	Species	Dilution	Source
AP-2	Mouse monoclonal	1:100	DSHB
ARL-13B	Rabbit polyclonal	1:100	Proteintech
BESTROPHIN	Mouse monoclonal	1:1000	Novus Biologicals
BRN3A	Mouse monoclonal	1:250	Merck/Millipore
CD18	Mouse monoclonal	1:100	Bio-Rad/Serotec
CONE ARRESTIN	Rabbit polyclonal	1:2000	Merck/Millipore
CRX	Mouse monoclonal	1:1000	Abnova
EZRIN	Mouse monoclonal	1:250	Sigma-Aldrich
GLUTAMIN SYNTHASE	Mouse monoclonal	1:500	Merck/Millipore
IBA1	Rabbit polyclonal	1 :100	WAKO
KI67	Mouse monoclonal	1:200	BD Pharmagen
LHX1 (LIM1)	Mouse monoclonal	1:20	DSHB
MITF	Mouse monoclonal	1:200	DAKO
OCT4	Rabbit monoclonal	1:100	Cell Signaling Tech.
OPSIN Blue	Rabbit polyclonal	1:500	Merck/Millipore
OPSIN R/G	Rabbit polyclonal	1:500	Merck/Millipore
PAX6	Rabbit polyclonal	1:1000	Merck/Millipore
PKCα	Rabbit polyclonal	1:5000	Santa-Cruz
RAX/RX	Rabbit polyclonal	1:5000	Abcam
RHODOPSIN	Mouse monoclonal	1:500	Merck/Millipore
RECOVERIN	Rabbit polyclonal	1:5000	Merck/Millipore
SSEA4	Mouse monoclonal	1:200	Cell Signaling Tech.
SOX2	Rabbit monoclonal	1:400	Cell Signaling Tech.
TRA1-81	Mouse monoclonal	1:100	Cell Signaling Tech.
VIMENTIN	Mouse monoclonal	1:200	Merck/Millipore
VSX2 (CHX10)	Goat polyclonal	1:2000	Santa Cruz
ZO1	Rabbit polyclonal	1:250	ThermoFischer Sci.

Table S2: List of TaqMan® Gene Expression ID Assays used for RT-qPCR

**Gene Symbols**	**Assays IDs (LifeTechnologies)**
18S	18S-Hs99999901_s1
BEST1	BEST1-00188249_m1
CRX	CRX-Hs00230899_m1
DKK1	DKK1-Hs00183740_m1
DNMT3B	DNMT3B-Hs00171876_m1
EN1	EN1- Hs00154977_m1
GAD2	GAD2-Hs00609534_m1
GDF3	GDF3-Hs00220998_m1
HAND1	HAND1-Hs00231848_m1
LHX2	LHX2-Hs00180351_m1
LIN28A	LIN28A-Hs00702808_s1
MERTK	MERTK-Hs01031973_m1
MITF	MITF-Hs01117294_m1
NANOG	NANOG-Hs02387400_g1
NEUROD1	NEUROD1-Hs00159598_m1
NKX2-1	NKX2-1- Hs00968940_m1
NODAL	NODAL-Hs00415443_m1
NOGGIN	NOG-Hs00271352_s1
NRL	NRL-Hs00172997_m1
PAX6	PAX6-Hs00240871_m1
PDEF	SERPINF1-Hs01106934_m1
POU5F1 (OCT4)	POU5F1-Hs00999632_g1
RAX	RAX-Hs00429459_m1
RPE65	RPE65-Hs01071462_m1
SIX3	SIX3-Hs00193667_m1
SOX2	SOX2-Hs01053049_s1
TDGF1	TDGF1- Hs02339497_g1
TERT	TERT-Hs00972656_m1
VSX2	VSX2-Hs00766959_s1
ZFP42	ZFP42- Hs01938187_s1
